# Hybrid genome assembly and comprehensive genomic analysis of *Lactiplantibacillus pentosus* LP309 reveal its probiotic and technological potential

**DOI:** 10.1186/s12864-026-12678-y

**Published:** 2026-02-24

**Authors:** Ana Marín-Gordillo, Elio López-García, Verónica Romero-Gil, Virginia Martín-Arranz, Francisco Noé Arroyo-López, Francisco Rodríguez-Gómez

**Affiliations:** 1 Oleica start-up (Technological Applications for Improvement of the Quality and Safety in Foods), Avenida Diego Martínez Barrio Nº10, Second Floor, Seville, 41013 Spain; 2 Instituto de la Grasa (CSIC), Food Biotechnology Department, Ctra. Sevilla-Utrera, km 1. Building 46. Campus Universitario Pablo de Olavide, Seville, 41013 Spain

**Keywords:** Whole-genome sequencing, Table Olives, Lactobacillus pentosus, Probiotic, Starter

## Abstract

**Background:**

This study provides an in-depth genomic and functional characterization of *Lactiplantibacillus pentosus* LP309, a strain isolated from table olive fermentations. The microorganism was sequenced using three next‑generation sequencing platforms—Illumina, PacBio, and Oxford Nanopore Technologies (ONT)—and multiple assembly and polishing strategies were evaluated. Assembly performance and annotation quality metrics were compared across approaches, and the most complete hybrid assembly for this study (Illumina + ONT) was selected for comprehensive genomic characterization.

**Results:**

The complete chromosome was circularized at 3,523,074 bp, and together with eight plasmids, the total genome length reached 3,743,370 bp, comprising 3,448 coding sequences (CDSs) and reflecting the genomic complexity of LP309 strain. Taxonomic assignment based on Average Nucleotide Identity confirmed the species identity, while functional annotation predicted the presence of two bacteriocin and two exopolysaccharide biosynthesis clusters. Additionally, over 150 genes related to the probiotic and technological potential of the strain were also identified, including those involved in adhesion, acid stress resistance, vitamin biosynthesis, and carbohydrate metabolism, among others. Safety assessment confirmed the absence of genes associated with virulence, antibiotic or acquired antimicrobial resistance. Mobilome analysis revealed four prophages, 133 insertion sequences, and four genomic islands, while no integrons were detected.

**Conclusions:**

This in silico study has revealed the strong technological relevance and probiotic potential of LP309, establishing this plant-based bacterium as a safe and functionally versatile candidate for applications in the food and biotechnology industries.

**Supplementary Information:**

The online version contains supplementary material available at 10.1186/s12864-026-12678-y.

## Background

Lactic acid bacteria (LAB) constitute a broad group of microorganisms belonging mainly to the order *Lactobacillales*, which encompasses several families of great technological and ecological relevance. These LAB are Gram-positive, facultatively anaerobic, non–spore-forming, hetero- or homofermentative, and acid-tolerant bacteria that produce lactic acid as the main metabolic end product from carbohydrate fermentation [1]. LAB can be found either as autochthonous or allochthonous microbiota, primarily in the mammalian gastrointestinal tract, but also on fresh fruits, vegetables and fermented food products [2].

Table olives are one of the most important fermented vegetables in Mediterranean basin, with a worldwide production exceeding 3 million tons per year [3]. *Lactiplantibacillus pentosus* (ex *Lactobacillus pentosus*) and *Lactiplantibacillus plantarum* (ex *Lactobacillus plantarum*) are among the predominant LAB species responsible for table olive fermentation, contributing to the flavor, quality, and safety of the final product [4]. Specifically, *L. pentosus* is a homofermentative LAB species that, in addition to table olives, has been also isolated from a variety of fermented foods such as pickles and cucumbers [5, 6]. The role of this species in vegetable fermentations is fundamental to ensure the quality and safety of the final product [4, 7]. For this reason, in recent years the ability of this LAB to serve as a starter culture for table olive fermentation, including industrial-scale processes, has been widely studied [8–10]. Specifically, Rodríguez-Gómez et al. [11] recently demonstrated that *L. pentosus* LP309 (hereinafter LP309) enhanced table olive fermentations when it was applied as starter, improving LAB survival during the initial stages and achieving lower pH values compared with fermentations conducted without this microorganism.

In addition, LAB are of particular interest for their probiotic potential, as specific strains from various genera can confer health benefits to the host when consumed in sufficient amounts [12, 13]. Specifically, research on the probiotic properties of *L. pentosus* has increased in recent years [14, 15], mainly due to the growing demand for plant-based probiotic products driven by a shift in consumer preferences toward healthier alternatives. Moreover, vegetarian trends and lactose intolerance have promoted the search for new dairy-free and vegan products. In this context, table olives are emerging as a promising plant-based probiotic source and a solid alternative to traditional dairy products [16].

Comprehensive genomic characterization of novel strains is crucial to evaluate their safety and functional potential for biotechnological applications, including their use as probiotics or starter cultures [17–19]. Recent advances in sequencing technologies, such as Illumina, PacBio, and Oxford Nanopore Technologies (ONT), enable high-quality genome assemblies, facilitating the precise identification of functional genes and mobile genetic elements [20, 21]. Therefore, this study aimed to apply a hybrid sequencing approach, integrating Illumina, PacBio, and ONT reads, to achieve an in-depth genomic characterization of LP309 and to predict its technological and probiotic potential through in silico analyses.

## Methods

### DNA extraction and sequencing

LP309 strain was previously isolated from Spanish-style table olive fermentations of the Manzanilla cultivar and identified through molecular analysis using multiplex *recA* gene PCR [22]. LP309 was grown in Man Rogosa and Sharpe broth (Oxoid, Basingstoke, UK) at 37 °C for 48 h prior to DNA extraction and isolation. Genomic and plasmid DNA were extracted and purified following the protocol described by Martín-Platero et al. [23]. Briefly, DNA was obtained using a modified salting-out procedure consisting of three steps: (i) cell resuspension in enzymatic buffer, (ii) bacterial lysis with lysis buffer followed by a 5 min incubation at 80 °C, and (iii) protein precipitation with a high-salt solution. DNA integrity was confirmed by visualization on 0.9% agarose gels, and its concentration was determined using a Qubit 4 fluorometer (Thermo Fisher Scientific, Waltham, USA).

Whole-genome sequencing of the strain was performed using Illumina HiSeq (FISABIO, Valencia, Spain), PacBio Sequel II (FISABIO, Valencia, Spain), and Oxford Nanopore MinION (Instituto de la Grasa, Seville, Spain). Illumina paired-end sequencing (2 × 150 bp) was carried out using Illumina sequencing-by-synthesis chemistry. PacBio high-fidelity (HiFi) circular consensus sequencing (CCS) reads were generated on a Sequel II platform using single-molecule real-time (SMRT) sequencing chemistry. Oxford Nanopore sequencing was performed using the Ligation Sequencing Kit V14 (SQK-LSK114) and R10.4.1 flow cells, following the manufacturer’s standard protocols. Basecalling of Nanopore reads was conducted using both fast and super-accurate (sup) modes, with the fast mode providing rapid basecalling at the expense of accuracy, and the super-accurate mode producing higher-accuracy reads at the cost of increased computational time. The raw reads from each platform were processed using Trim Galore v0.6.10 [24], fastp v1.0.1 [25], and Porechop v0.2.4 [26], respectively. Read quality was evaluated using FastQC v0.12.1 [27], MultiQC v1.27 [28], and NanoPlot v1.44.1 [29].

### Genome assembly

This analysis was carried out using different strategies. For short Illumina reads, SPAdes v4.2.0 [30] and Unicycler v0.5.1 [31] were tested. Long-read assemblies were generated using Flye v2.9.6 [32] and Unicycler with PacBio and ONT reads. Hybrid assemblies combining short and long reads (Illumina+PacBio and Illumina + ONT) were performed with Unicycler. Assembly quality metrics included the number of contigs, length of the longest contig, total genome size, N50, L50, and GC content. Completeness was further assessed by searching for conserved orthologous genes using BUSCO v5.8.0 [33] with two databases: bacteria_odb10, which contains orthologs conserved across all bacteria, and lactobacillales_odb10, specific for the order *Lactobacillales*. Rapid structural and functional annotation was performed using Prokka v1.14.16 [34], and annotation quality was evaluated based on the number of predicted genes and RNAs. Sequencing reads and the complete genome assembly were deposited in the European Nucleotide Archive (ENA) under Bioproject accession number PRJEB94968.

### Taxonomic identification and phylogenetic classification

To verify the phylogenetic classification of LP309, an Average Nucleotide Identity (ANI) analysis was performed, including several strains of *L. plantarum*, *L. paraplantarum*, *L. pentosus*, and other genera within the *Lactobacillaceae* family (see Table S1, supplementary material). ANI analysis was performed using a Python script [35], and clustering with heatmap visualization was generated using the pheatmap package in R. Moreover, to evaluate gene alignment and synteny between *L. pentosus* LP309 and other *L. pentosus* strains, comparative genomic analyses were performed using the progressiveMauve algorithm [36]. The reference genome used was *L. pentosus* DSM 20,314, together with strains isolated from table olive fermentations (*L. pentosus* MP-10 and *L. pentosus* LPG1) and from cucumber fermentation (*L. pentosus* 1.8.9).

### Safety assessment

The genomic safety of LP309 was assessed using multiple bioinformatics tools. First, the Comprehensive Antibiotic Resistance Database (CARD) and the Resistance Gene Identifier (RGI) were used to identify antibiotic resistance genes [37]. Second, acquired antimicrobial resistance (AMR) genes were screened with ResFinder v4.0 [38], using thresholds of 80% identity and 60% minimum length. In addition, PathogenFinder v1.1 [39] and VirulenceFinder v2.0 [40] were used to predict potential human pathogenicity and to identify acquired virulence genes, respectively.

### Functional genome annotation and identification of key genes

A comprehensive functional annotation of the LP309 genome was performed using eggNOG-mapper v5.0 [41], providing the number and distribution of genes across COG functional categories. dbCAN3 [42] was used for the specific annotation of genes involved in carbohydrate metabolism. Genes coding for bacteriocins and ribosomally synthesized and post-translationally modified peptides (RiPPs) were predicted using the BAGEL4 database [43], while antiSMASH v6.0 [44] was used to identify secondary metabolite gene clusters. Additionally, genes associated with probiotic and technological traits—including adhesion, tolerance to acid and bile salts, gut microbiota modulation, exopolysaccharide (EPS) and vitamin biosynthesis, as well as phenolic and carbohydrate degradation—were manually curated. The complete LP309 genome was screened using BLASTp against amino acid sequences of target genes, applying thresholds of 50% identity, 70% query coverage, and an E-value of 1E − 9. Genes detected by BLAST but not annotated by Prokka or eggNOG were subsequently analyzed with UniProt BLAST, as this database contains numerous manually-curated entries. CRISPR and CRISPR-associated (Cas) genes were identified using CRISPRCasFinder v1.1.2-I2BC [45], and CRISPR arrays were confirmed according to the CRISPRdb database. A circular genomic map of the LP309 chromosome was generated using Proksee [46].

### Mobilome analysis and plasmid identification

Prophage regions were predicted using PHASTEST [47], and ISEScan version 1.7.3 [48] was used to identify insertion sequences (IS) and transposons. Genomic islands (GIs) were detected using IslandViewer4 [49], and IntegronFinder version 2.0.5 [50] was employed to search for integrons. For plasmid identification, the hybrid assembly (Illumina + ONT) was analyzed with RFPlasmid version 1.0 [51]. Contigs identified as plasmids were further compared using BLAST against plasmids from phylogenetically related strains.

## Results

### Sequencing results and genome assembly comparison

The results obtained from the different sequencing technologies are summarized in Table 1. Illumina sequencing of the *L. pentosus* LP309 genome generated a total of 10,748,663 reads with an average read length of 149.5 bp. In contrast, long-read sequencing platforms produced a lower number of reads, with 100,681, 234,372, and 233,486 reads obtained from the PacBio platform, ONT using fast basecalling, and ONT using sup basecalling, respectively. However, the average read length was considerably higher for long-read technologies, reaching 3,914.1 bp, 2,764.2 bp, and 2,786.2 bp for PacBio, ONT fast basecalling, and ONT sup basecalling, respectively. Illumina sequencing yielded the highest total number of sequenced bases and the highest estimated coverage, as shown in Table 1.

**Table 1:** Summary of sequencing output and coverage across different sequencing platforms

Platform	Reads number	Average Raw Read Length (bp)	Nº bases (GB)	Estimated coverage (x)
Illumina	10,748,663	149.5	3.2	859.7
PacBio	100,681	3914.1	0.39408	105.3
ONT fast basecalling	234,372	2764.2	0.64785	173.1
ONT sup basecalling	233,486	2786.2	0.65055	173.8

The results of genome assemblies using the different sequencing reads, their combinations, and the various assembly software are summarized in Table 2. As expected, the highest number of contigs was obtained with assemblies generated exclusively from Illumina reads, with 198 contigs using Unicycler and 283 contigs using SPAdes. Conversely, the lowest number of contigs (9) was obtained in hybrid assemblies, both Illumina + PacBio and Illumina + ONT. Assemblies generated solely from PacBio reads produced a higher number of contigs with both assemblers than those obtained from ONT reads, regardless of whether the fast or sup basecalling mode was used. As expected, a lower number of contigs correlated with a longer maximum contig length, which ranged from 156,631 bp for the Illumina + Unicycler assembly to 3,523,077 bp for the Illumina + PacBio hybrid assembly. Genome size also varied depending on the type of reads and the assembler used, although the differences were less pronounced than for the maximum contig length, ranging from 3,539,667 bp (Illumina + Unicycler) to 3,830,536 bp (ONT SUP + Unicycler). As shown in Table2, N50 and L50 metrics also varied according to contig length distribution. The GC content of the assemblies ranged from 45.99% (PacBio + Flye and ONT + Unicycler) to 46.29% (Illumina + Unicycler).

**Table 2:** Assembly statistics for different sequencing read combinations: ONT (Oxford Nanopore Technology), I + PB (Illumina + PacBio), and I + ONT (Illumina + Oxford Nanopore Technology)

Platform	Assembler	Polishing	Number of Distinct Contigs	Largest Contig (bp)	Genome Length (bp)	N50	L50	G/C (%)
Illumina	Unicycler	-	198	156,631	3,539,667	48,298	23	46.29
	SPAdes	-	283	156,793	3,616,829	46,246	25	46.17
PacBio	Unicycler	Racon	12	2,085,526	3,740,971	2,085,526	1	46.06
	Flye	Racon	17	2,134,709	3,698,001	2,134,709	1	45.99
ONT fast basecalling	Unicycler	Medaka	10	3,511,591	3,817,160	3,511,591	1	45.99
	Flye	Medaka	10	3,513,390	3,759,657	3,513,390	1	46.09
ONT sup basecalling	Unicycler	Medaka	10	2,156,321	3,830,536	2,156,321	1	46.08
	Flye	Medaka	10	3,519,984	3,757,627	3,519,984	1	46.10
I + PB	Unicycler	-	9	3,523,077	3,743,278	3,523,077	1	46.11
I + ONT	Unicycler	-	9	3,523,074	3,743,370	3,523,074	1	46.11

**Fig. 1:**
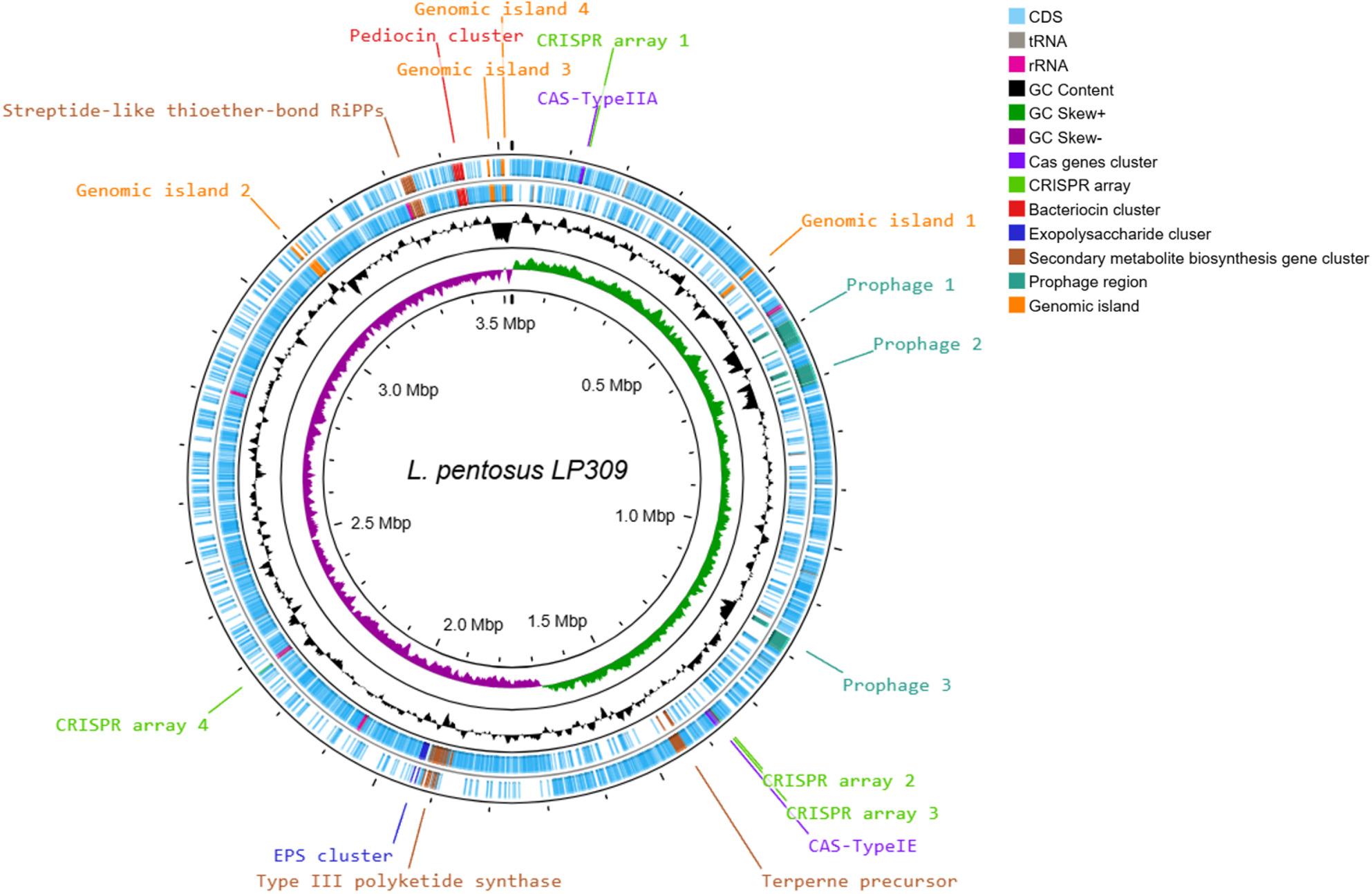
Circular map of the L. pentosus LP309 chromosome. The outermost and second rings display coding sequences (CDSs) and important markers (labels) in the forward strand and reverse strand respectively. The third ring (black) shows GC content variation, followed by the GC skew profile. Genomic coordinates are represented in the innermost ring

Assembly completeness was also evaluated using BUSCO, comparing the prediction of conserved orthologous genes at both the bacterial level (bacteria_odb10) and the *Lactobacillales*-specific level (lactobacillales_odb10) across all assemblies (Table 3). For conserved bacterial orthologs, most assemblies obtained 123/124 complete BUSCO genes, except for assemblies generated from ONT fast basecalling, which showed a reduced number of complete BUSCOs: 94/124 (Unicycler) and 95/124 (Flye). Missing BUSCO genes in these assemblies were classified as either fragmented or absent. For *Lactobacillales*-specific orthologs, most assemblies achieved a perfect score of 402/402 complete BUSCO genes, except for the ONT fast basecalling assemblies, which yielded 313/402 (Unicycler) and 321/402 (Flye). Rapid genome annotation using Prokka predicted a total gene count ranging from 3,225 genes (Illumina + Unicycler) to 4,373 genes (ONT fast + Unicycler). In contrast, the number of predicted rRNA genes was lower in assemblies derived exclusively from Illumina short reads compared with assemblies that included long reads. Complete annotation metrics are provided in Table 4. Fig. 1 shows also a complete circular genomic map of the LP309 chromosome.

**Table 3:** BUSCO gene identification results for bacterial and Lactobacillales conserved orthologs

			BUSCO Bacteria				BUSCO Lactobacillales			
Platform	Assembler	Polishing	BUSCO genes complete	Duplicated BUSCO	BUSCO genes Fragmented	BUSCO genes Missing	BUSCO genescomplete	Duplicated BUSCO	BUSCO genes Fragmented	BUSCO genes Missing
Illumina	Unicycler	-	123/124	1	1	0	402/402	1	0	0
	SPAdes	-	123/124	1	1	0	402/402	1	0	0
PacBio	Unicycler	Racon	123/124	2	1	0	402/402	5	0	0
	Flye	Racon	123/124	1	1	0	402/402	1	0	0
ONT fast basecalling	Unicycler	Medaka	94/124	0	23	7	313/402	1	48	41
	Flye	Medaka	95/124	0	23	6	321/402	1	47	34
ONT sup basecallig	Unicycler	Medaka	123/124	1	1	0	402/402	1	0	0
	Flye	Medaka	123/124	1	1	0	402/402	1	0	0
I+PB	Unicycler	-	123/124	1	1	0	402/402	1	0	0
I+ONT	Unicycler	-	123/124	1	1	0	402/402	1	0	0

**Table 4:** Summary of rapid annotation and rRNA gene identification across read combinations for unique and hybrid assemblies

Platform	Assembler	Polishing	Annotator	Numbers of Genes	Number of RNAr Operons
Illumina	Unicycler	-	PROKKA	3,289	5
	SPAdes	-		3,225	4
PacBio	Unicycler	Racon		3,528	16
	Flye	Racon		3,551	11
ONT fast basecalling	Unicycler	Medaka		4,373	16
	Flye	Medaka		4,208	16
ONT sup basecallig	Unicycler	Medaka		3,536	16
	Flye	Medaka		3,448	16
I+PB	Unicycler	-		3,443	16
I+ONT	Unicycler	-		3,448	16

### ANI and genome synteny analysis

**Fig. 2:**
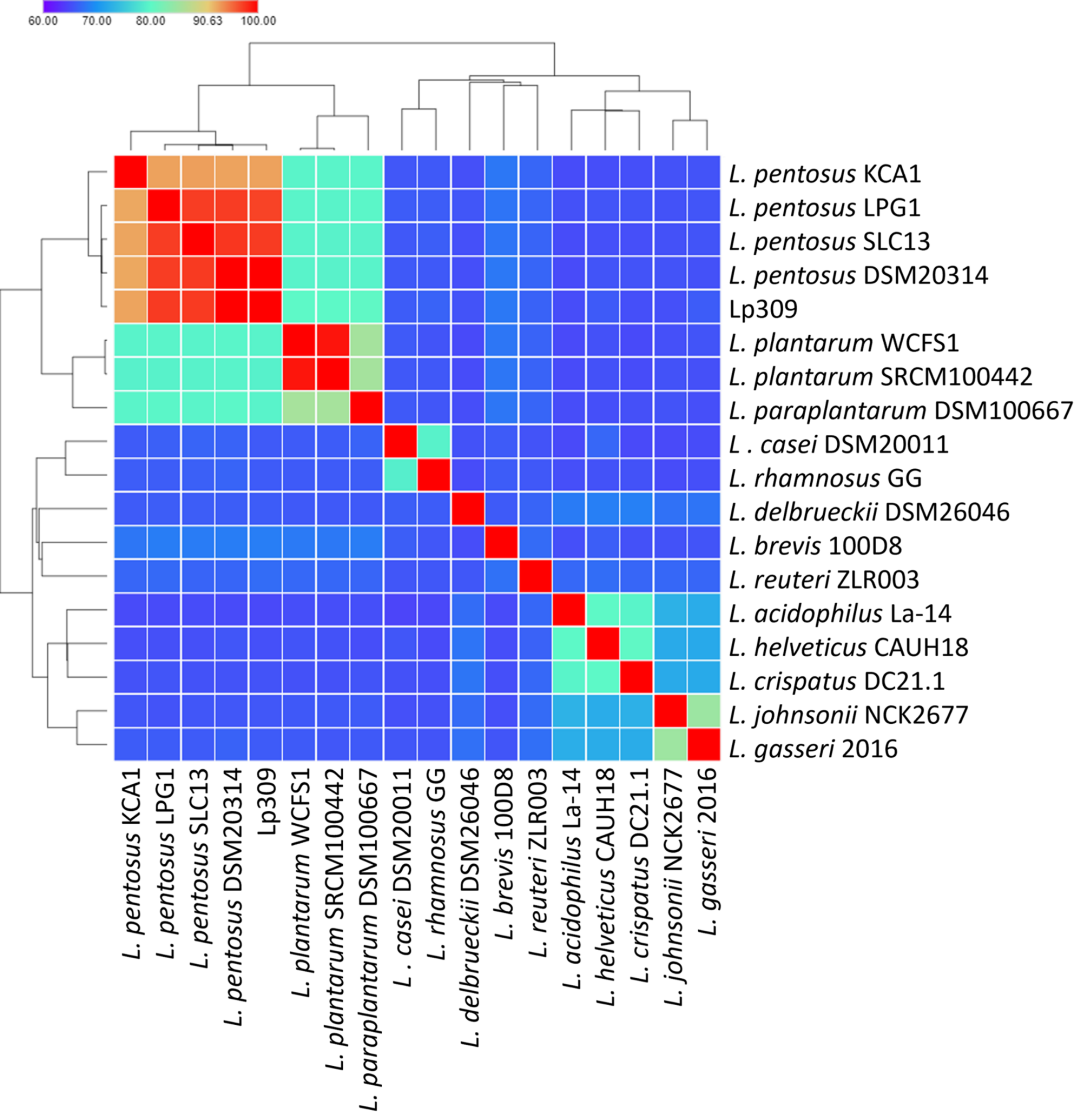
Hierarchical clustering and heatmap based on ANI, illustrating the genomic relatedness between LP309 and representative strains of L. pentosus, L. plantarum, L. paraplantarum, and other members of the Lactobacillaceae family

**Fig. 3:**
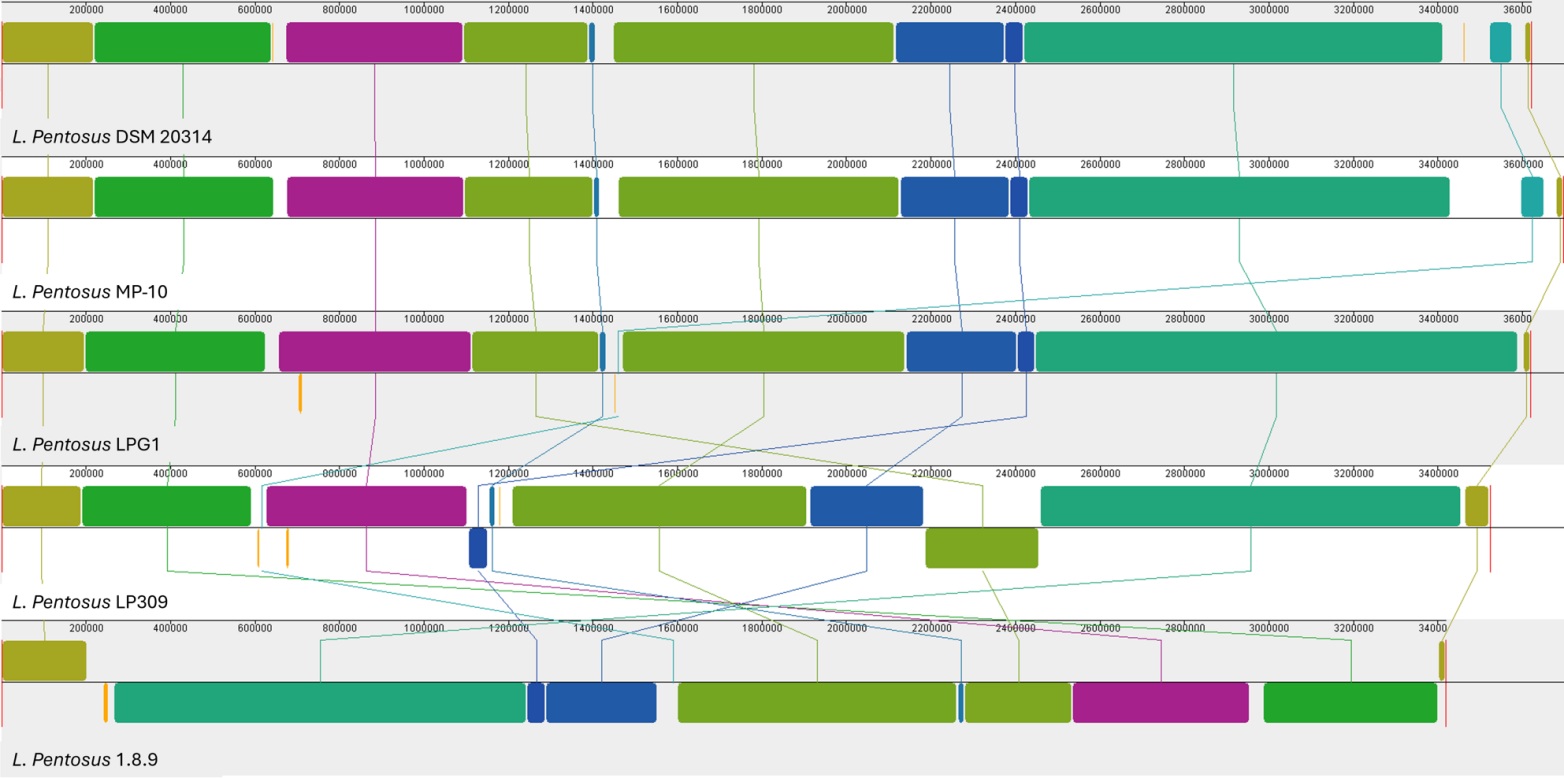
Mauve visualization of whole genome alignment of L. pentosus LP309 with L. pentosus DSM 20314, L. pentosus MP-10, L. pentosus LPG1 and L.pentosus 1.8.9

ANI analysis confirmed that LP309 strain belongs to the species *L. pentosus*, showing a similarity of more than 96% with the 4 reference genomes of *L. pentosus* used. According to the ANI analysis, LP309 strain exhibited the highest identity value (97.43%) with strain *L. pentosus* DSM 20,314 (Table S2). The strain was clearly differentiated from other genera within the *Lactobacillaceae* family, particularly from its closest phylogenetic neighbors *L. plantarum* and *L. paraplantarum* (Fig. 2). Genome synteny analysis revealed that the overall genomic organization of *L. pentosus* LP309 was largely conserved when compared with the reference strain *L. pentosus* DSM 20,314 and the table-olive-associated strain *L. pentosus* MP-10 and *L. pentosus* LPG1. However, several genomic regions exhibited inversions and rearrangements. Notably, some of these rearranged regions were located in positions more similar to those observed in strains isolated from other fermentative niches, such as *L. pentosus* 1.8.9 from cucumber fermentation. (Fig. 3).

### Safety assessment

No acquired antibiotic or antimicrobial resistance genes were detected, and no virulence or pathogenicity factors were identified in the screened databases. Consistently, the PathogenFinder tool classified strain LP309 as non-pathogenic.

### Functional genome analysis

**Fig. 4:**
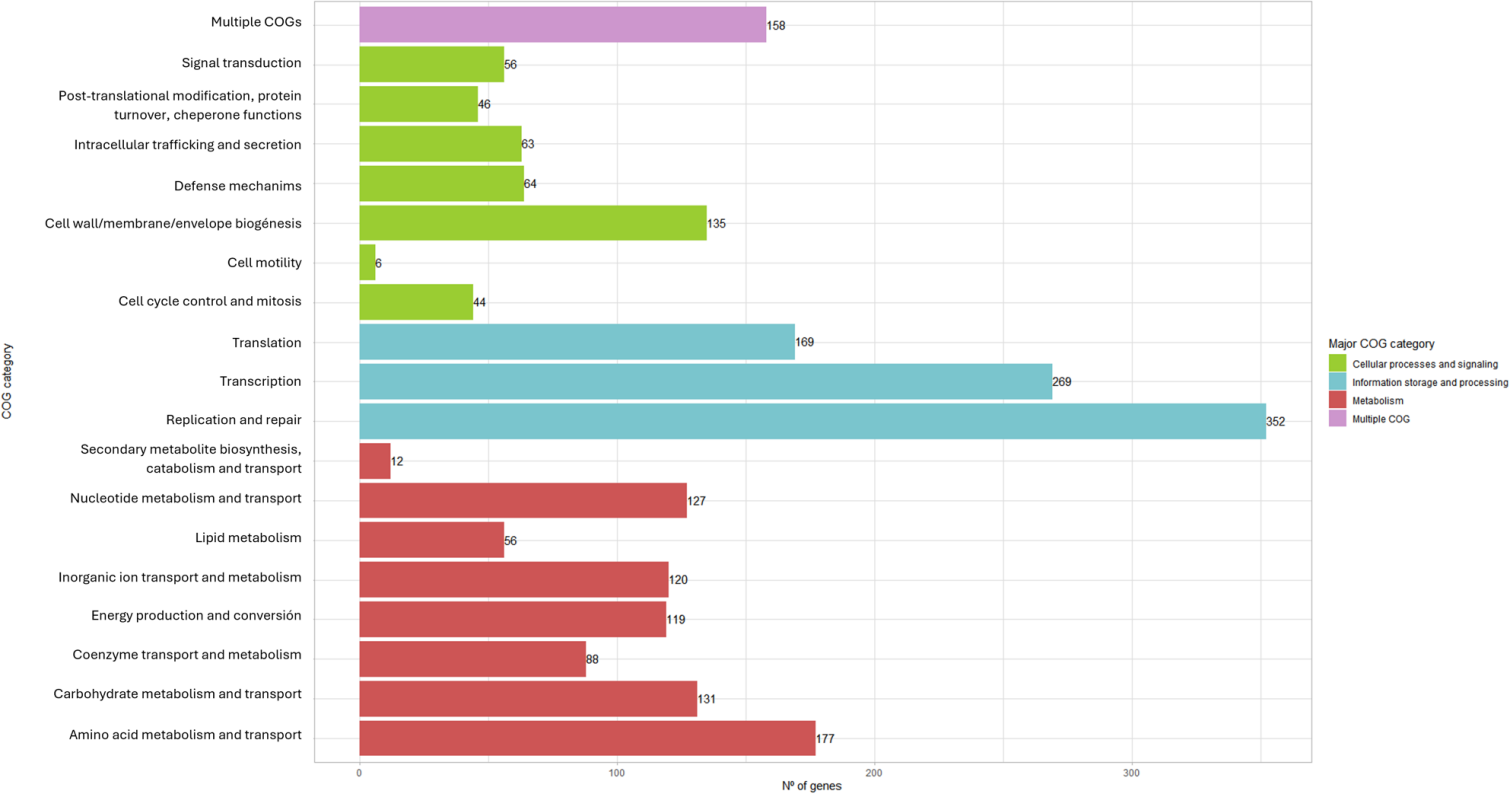
Functional distribution of LP309 predicted coding sequences based on COG (Clusters of Orthologous Groups) categories, annotated using EggNOG-mapper. Genes of unknown function or lacking COG assignment are excluded from the chart

The functional distribution of genes within the LP309 genome across COG categories is shown in Fig. 4. The category with the highest number of genes was replication and repair (352 genes), followed by transcription (269), amino acid metabolism, and transport (177). A total of 81 genes involved in carbohydrate metabolism were identified with high confidence according to the dbCAN3 server, including 36 glycoside hydrolases (GHs), 33 glycosyltransferases (GTs), 8 carbohydrate-binding modules (CBMs), and 3 carbohydrate esterases (CEs). Two putative bacteriocin biosynthetic clusters were predicted, with a pediocin cluster located on the chromosome and a bovicin 255 variant cluster on contig 4. Regarding secondary metabolism, several functional regions were predicted, including a terpene precursor, a type III polyketide synthase, and a streptide-like thioether-bound RiPP cluster. The chromosomal location of these regions is shown in Fig. 1. In addition, 158 genes associated with probiotic and technological potential were predicted in LP309 strain (see Table S3, supplementary material). These included 22 genes related to bacterial adhesion, 36 involved in acid and bile salt resistance, 6 in intestinal microbiota modulation, 32 in vitamin biosynthesis, 27 in exopolysaccharide (EPS) production, 31 in carbohydrate metabolism, and 9 in phenolic compound degradation.

### Mobilome and plasmid characterization

**Fig. 5:**
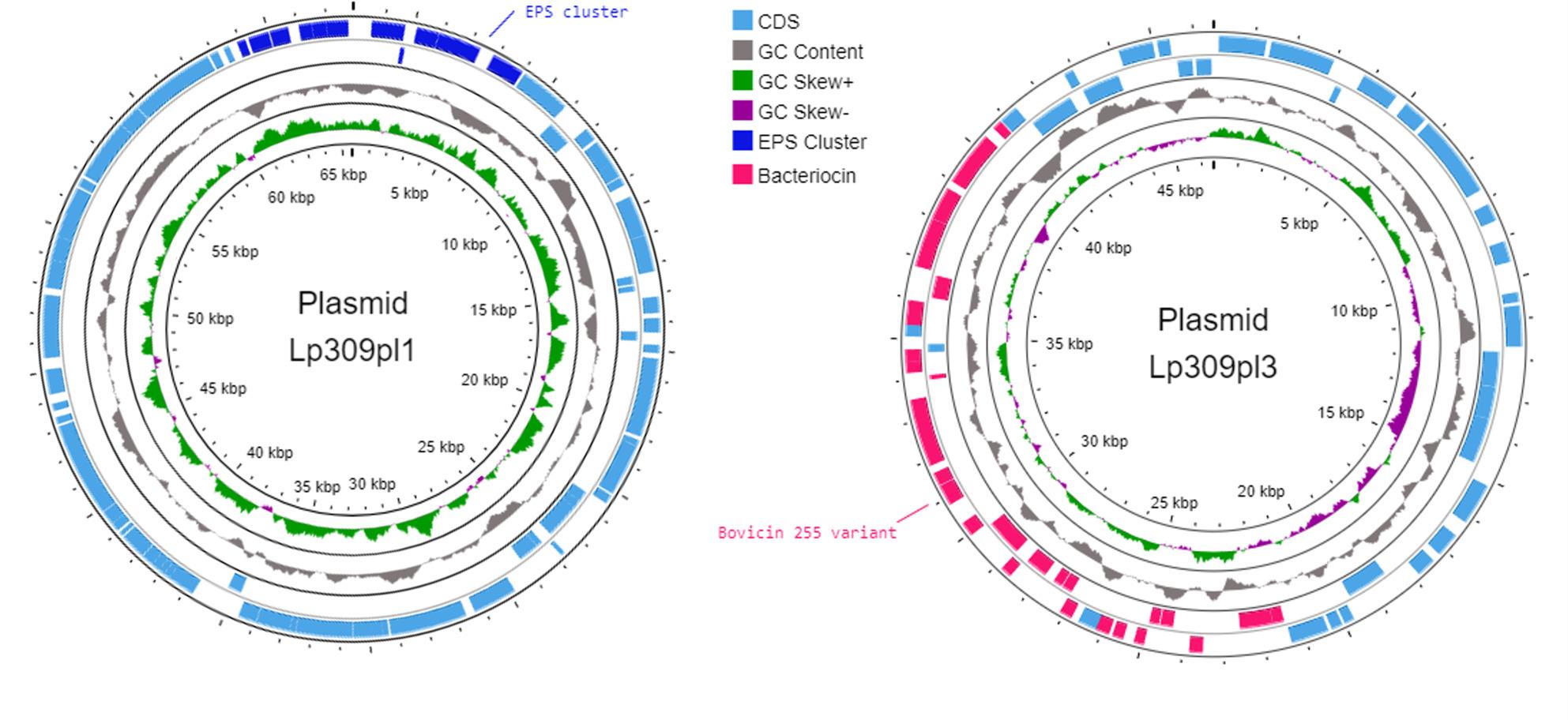
Circular map of plasmids 1 and 3 from L. pentosus LP309. The outermost and second rings display coding sequences (CDSs) and important markers (labels) in the forward strand and reverse strand respectively. The third ring (black) shows GC content variation, followed by the GC skew profile. Genomic coordinates are represented in the innermost ring

The mobilome refers to the set of mobile genetic elements within a genome, including prophages, IS, genomic islands, integrons, and plasmids. For LP309 strain, a total of 3 intact prophages were predicted in the chromosome using PHASTEST, with lengths of 44.6 kb, 37.1 kb, and 38.5 kb. In all 3 prophages, essential proteins were identified, supporting their presence in the chromosome, including genes encoding integrase, terminase, portal protein, protease, head and capsid proteins, tail proteins, and lysin. Additionally, two questionable prophages were detected, although their presence could not be fully confirmed. The genomic positions of these prophages are shown in Fig. 1. A total of 133 IS were predicted across the genome, including both the chromosome and plasmids. Furthermore, 4 genomic islands (GIs) were identified, whose positions are also displayed in Fig. 1, while no integrons were predicted in the LP309 genome. Finally, a total of 8 plasmids were identified in LP309. The best-scoring plasmid matches after BLAST comparison against the NCBI plasmid database are listed in Table 5. The majority of the BLAST results exhibited alignment coverage with database plasmids exceeding 70%, and in all cases, sequence identity was above 95%. The most important plasmids were graphed and are shown in Fig. 5.

**Table 5:** BLAST results of plasmids identified using RFplasmid from the genome of *L. pentosus* LP309

seq.ID	Plasmid Size (bp)	Number of Genes	Accession Number	Organism/Strain	Query Coverage (%)	Identity (%)
LP309_contig_2	65,557	67	CP151093.1	*L. plantarum* strain L75 plasmid p_no.1	100	99.99
LP309_contig_3	60,573	59	CP099564.1	*L. pentosus* strain KW2 plasmid p6	56	99.72
LP309_contig_4	46,489	53	CP099985.1	*L. plantarum* strain 3 − 1 plasmid pLP314	71	99.24
LP309_contig_5	17,521	19	CP168272.1	*L. plantarum* strain JY067 plasmid unnamed2	75	99.82
LP309_contig_6	13,601	16	CP166735.1	*L. plantarum* strain HD02 plasmid pHD02D	79	98.66
LP309_contig_7	8,392	10	CP134793.1	*L. pentosus* strain 7.8.2 plasmid unnamed	53	99.86
LP309_contig_8	6,348	7	CP183366.1	*L. plantarum* strain ZG308 plasmid unnamed6	57	94.98
LP309_contig_9	1,815	2	CP063752.1	*L. plantarum* strain ATCC 202,195 plasmid unnamed2	100	99.89

The use of a hybrid assembly approach allowed the estimation of the relative copy number of each plasmid present in the population at the time of DNA extraction from *L. pentosus* LP309, by normalizing plasmid coverage to chromosomal coverage. As shown in Table 6, several plasmids were present at approximately one copy per chromosome, including plasmids LP309pl1, LP309pl2 and LP309pl3. Plasmids LP309pl4, LP309pl5, LP309pl6, and LP309pl8 were detected at relative abundances ranging from 2 to 7 copies per chromosome. In contrast, LP309pl7 plasmid was present at a markedly higher abundance, with an estimated 29 copies per chromosome within the clonal population. In addition, the replication and mobilization potential of each plasmid was further investigated through identification of genes involved in these processes, in order to assign each plasmid to a putative replicon class (Table S4). Only plasmid LP309pl1 was predicted to encode a conjugation-related protein (LP309_03358, conjugal transfer protein TraG) and was therefore classified as potentially conjugative. Plasmids LP309pl2, LP309pl3, LP309pl4, LP309pl5, and LP309pl7 were predicted to encode proteins involved in plasmid mobilization and were thus considered putatively mobilizable. In contrast, plasmids LP309pl6 and LP309pl8 were classified as non-mobilizable due to the absence of genes encoding proteins associated with plasmid transfer. The estimated copy number of each plasmid, together with its predicted replicon type based on replication and mobilization gene content, is summarized in Table 6.

**Table 6:** Overview of plasmid copy number and replicon classes in the LP309 genome

Plasmid Name	Copy number	Mobility	Replicon class
LP309pl1	1.76	Conjugative	Potentially conjugative plasmid
LP309pl2	1.87	Mobilizable	Putatively mobilizable plasmid
LP309pl3	1.67	Partial	Putatively mobilizable plasmid
LP309pl4	2.17	Mobilizable	Putatively mobilizable plasmid
LP309pl5	4.17	Mobilizable	Putatively mobilizable plasmid
LP309pl6	3.21	Non mobilizable	Non-mobilizable plasmid
LP309pl7	29.14	Mobilizable	Putatively mobilizable plasmid
LP309pl8	7.35	Non mobilizable	Non-mobilizable plasmid

## Discussion

Thus far, most studies comparing genome assemblies generated from different sequencing technologies reads have focused primarily on well-characterized species such as *Escherichia coli*, *Klebsiella pneumoniae*, and *Mycobacterium tuberculosis* [20, 52, 53], or on less complex bacterial genomes such as *Streptococcus pyogenes* or *Campylobacter jejuni* [54, 55], whose genome sizes typically do not exceed 2 Mb. In this work, we compare assemblies produced by different assembly tools and using reads from various sequencing technologies for a complex, environmentally derived genome, *L. pentosus* LP309. As described previously, the hybrid assemblies generated using Illumina + ONT and Illumina+PacBio with Unicycler were the most complete, comprising 9 contigs with an N50 greater than 3.5 Mb, a GC content of 46.11%, and a full recovery of all *Lactobacillales* BUSCO genes. The ONT assembly using sup basecalling mode with Flye produced comparable results, whereas Illumina short reads alone generated highly fragmented assemblies (> 198 contigs). These findings confirm that hybrid assembly remains as the most reliable approach for generating complete bacterial genomes [53, 56], particularly when accounting for the higher intrinsic error rates historically associated with long-read sequencing technologies. However, recent improvements in ONT pore chemistry and basecalling algorithms have substantially narrowed this gap, allowing long-read-only assemblies to approach similar levels of completeness [54]. Furthermore, the comparison between fast and sup basecalling modes in ONT demonstrated that the fast mode introduces a higher error rate than the high-accuracy mode [57]. The number of annotated genes ranged from 3,225 in Illumina-only assemblies to 4,373 in ONT fast basecalling assemblies, with hybrid assemblies showing a more consistent prediction of approximately 3,448 genes. The inflated gene count observed in ONT fast basecalling assemblies is primarily attributed to nucleotide errors that introduce premature stop codons, leading to gene fragmentation and artificial gene duplication during annotation. In contrast, the use of sup basecalling, together with subsequent polishing, significantly mitigated these errors, resulting in more accurate and biologically consistent gene predictions [57]. Regarding rRNA gene recovery, assemblies generated from long reads alone or hybrid approaches consistently detected all rRNA genes, whereas assemblies based solely on short reads failed to recover complete rRNA operons. This limitation is inherent to the short length of Illumina reads, which cannot span the full rRNA gene regions [55]. Based on these results, the Illumina + ONT hybrid assembly generated with Unicycler was finally selected as the reference assembly for *L. pentosus* LP309.

In recent years, ANI analysis has been widely adopted as the genomic standard equivalent to the traditional DNA–DNA hybridization (DDH) method for the taxonomic classification of closely related bacterial species, particularly within the *Lactobacillaceae* family. As previously noted by Torriani et al. [22], *L. pentosus* and *L. plantarum* cannot be reliably distinguished by 16 S rRNA gene sequencing alone. Accurate phylogenetic resolution requires *recA* gene amplification or, alternatively, whole-genome sequencing (WGS). In the case of LP309 strain, the ANI analysis (Fig. 2) fully confirmed its previously assigned taxonomic identity. ANI analysis has also been used to confirm the identity of other *L. pentosus* strains such as L33, LPG1, or TBRC 20,328 [19, 58, 59]. Comparative genomic analyses indicate that genome rearrangements, particularly inversions and translocations, drive differences in gene order across bacterial genomes [60]. In this study, synteny analysis across multiple *L. pentosus* genomes corroborated this pattern (Fig. 3), revealing that LP309 exhibits a higher degree of syntenic conservation with strains isolated from table olive fermentations than with strains derived from other plant-based fermentations, such as cucumber. This observation is consistent with the possibility that niche-specific selective pressures may influence the long-term genomic organization of *L. pentosus* strains.

*L. pentosus* holds Qualified Presumption of Safety (QPS) status from the European Food Safety Authority (EFSA). The safety of the genus *Lactiplantibacillus* has also been demonstrated repeatedly in the literature [61, 62]. However, previous studies have reported that some *Lactobacillus* strains harbor antimicrobial resistance (AMR) genes, including resistance mechanisms to chloramphenicol, tetracyclines, β-lactams, and aminoglycosides [63]. The in silico safety assessment of LP309 revealed the presence of a single antibiotic resistance gene, specifically vanY from the vanB cluster, which is typically associated with vancomycin resistance. However, no other vancomycin resistance genes were detected. Vancomycin resistance in lactobacilli is considered intrinsic and non-transferable, and therefore not a safety concern according to EFSA guidelines. These results were consistent with other *L. pentosus* strains, such as L33, LPG1, or CF2-10 N [19, 58, 64].

Functional annotation of the LP309 genome revealed a high proportion of genes involved in DNA replication and repair, along with genes related to transcription, translation, and amino acid metabolism. Similar distributions were reported by Stergiou et al. [58] for the *L. pentosus* strain L33 and across 26 additional *L. pentosus* strains, suggesting a high degree of genomic stability and efficient gene expression. Conversely, LP309 harbors fewer genes related to carbohydrate metabolism and transport than other *L. pentosus* strains isolated from plant-based fermentations [19]. Nevertheless, LP309 performs efficiently in table olive fermentation, suggesting a specialized sugar metabolism adapted to the carbohydrates naturally present in this type of plant-based fermentation [11].

Focusing on carbohydrate-active enzymes (CAZy), the predicted gene distribution included 36 glycoside hydrolases (GHs), 33 glycosyltransferases (GTs), 8 carbohydrate-binding modules (CBMs), and 3 carbohydrate esterases (CEs). These results were consistent with other *L. pentosus* genomes [18, 58] but revealed a higher CAZy content compared to other *Lactobacillaceae* species, such as *L. casei*, *L. rhamnosus*, and *L. helveticus* [65]. This pattern likely illustrates the metabolic versatility of *L. pentosus*, which enables adaptation to various environments and efficient utilization of a wide range of carbohydrates as carbon sources. Moreover, LP309 harbored genes implicated in the degradation of complex carbohydrates, including starch, glycogen, oligosaccharides, cellulose, and xylans (Table S3, supplementary material), consistent with its ability to colonize heterogeneous plant-based niches [66]. In contrast, species like *L. casei* and *L. rhamnosus* are more typically associated with host-adapted environments [67].

Bacteriocins, antimicrobial peptides produced by bacteria, were also identified in LP309. Two bacteriocin-associated loci were identified in silico. The first, a pediocin-like region located on the chromosome, was initially classified as a Class IIa bacteriocin cluster, which typically targets *Listeria monocytogenes*, *Clostridium perfringens*, and certain *Enterococcus* and *Lactobacillales* strains [68]. Pediocin-like bacteriocins are widely distributed in *L. pentosus* and are also frequently reported in *L. plantarum* [69]. However, manual curation revealed that this pediocin-like region lacks the core biosynthetic genes encoding the prebacteriocin peptide, as well as the dedicated transport machinery required for class IIa pediocin production, indicating that this locus is incomplete. A second bacteriocin-associated region, predicted by BAGEL4 as a bovicin 255–like locus, was identified on contig 4. Bovicin 255 is a Class I lantibiotic primarily active against Gram-positive bacteria, including *L. monocytogenes*, *Clostridium* spp., and *Enterococcus* spp [68]. Although bovicin is most commonly associated with *Streptococcus* species, its presence in *L. pentosus* has also been reported [70]. Nevertheless, manual inspection of the predicted bovicin-like region revealed the absence of the core biosynthetic genes required for lantibiotic production, suggesting that this locus represents a partial or degenerate bacteriocin-associated region rather than a complete biosynthetic gene cluster. Additionally, several putative biosynthetic gene clusters (BGCs) were identified, including those predicted to produce terpene precursors, type III polyketide synthases, and streptide-like thioether-bond RiPPs. These BGCs are key elements for the synthesis of antimicrobial compounds [71, 72]. Collectively, these genomic features suggest that LP309 is a strain with strong antimicrobial potential and notable adaptability to plant-based fermentation niches, enhancing its survival and competitiveness in such environments.

Numerous genes involved in the technological and probiotic potential of the strain were predicted and are shown in Table S3 (Supplementary material). Among the adhesion-related genes, several genes encoding proteins with Mucus Binding-Protein domains were identified, as well as genes encoding cell surface proteins with LPXTG motifs (Table S3), suggesting a certain ability to adhere to both biotic and abiotic surfaces [73, 74]. Genes encoding multifunctional enzymes such as enolase and glyceraldehyde-3-phosphate dehydrogenase were also identified, with roles extending beyond metabolism to include adhesion [18, 75].

In addition, 3 genes encoding choloylglycine hydrolase, an enzyme involved in bile salt resistance, were predicted in LP309, which may enable survival in the gastrointestinal environment [76]. Several other genes associated with acid resistance were also identified, including *clpC*, *groL*, *groS*, *dnaJ*, and *dnaK*, which encode proteases and chaperones, indicating a good tolerance to acid and heat stress [77, 78]. Furthermore, the presence of multiple copies of stress-response genes such as *sasA* and *uvrA* reinforces the ability of the strain to cope with adverse conditions [79].

Regarding microbiota modulation, the LP309 strain carries 3 copies of the gene encoding D-lactate dehydrogenase and 3 additional copies encoding L-lactate dehydrogenase, both enzymes involved in lactic acid production. In the intestinal environment, lactic acid may synergize with butyrate-producing colonic bacteria, which convert lactic acid into butyric acid, a compound with well-established anti-inflammatory benefits for the gut epithelium [80].

In terms of vitamin biosynthesis, genome annotation predicted complete pathways for the production of riboflavin (vitamin B2) and folate (vitamin B9). Therefore, under appropriate conditions and with sufficient precursors, the strain could synthesize both vitamins, conferring an additional probiotic potential to LP309 [79]. Other biosynthetic pathways, such as thiamine (B1), pantothenic acid (B5), vitamin B6 (pyridoxal P) and vitamin K2 (menaquinone) were incomplete.

Moreover, functional annotation predicted 2 complete clusters for EPS production, which could contribute to biofilm formation and enhance intestinal persistence [81]. EPS produced by members of the *Lactobacillaceae* family has also been associated with the inhibition of certain pathogens [82] and modulation of the immune system [83, 84]. Rodríguez-Gómez et al. [11] previously reported the ability of this strain to produce EPS during table olive fermentations.

Finally, the genome revealed several genes encoding proteins involved in phenolic compound metabolism, including lipases, esterases, tannases, and phenolic acid decarboxylases (Table S3). This is of particular interest for LP309, which was isolated from table olive fermentations where phenolic compounds, known for their antimicrobial activity, are present at measurable concentrations [85]. Genes encoding proteins involved in the metabolism of phenolic compounds have also been reported in other *L. pentosus* strains [19, 86]. Therefore, this repertoire of genes likely contributes to the tolerance of LP309 to phenolic-rich environments.

CRISPR-Cas systems provide adaptive immunity in prokaryotes by preventing the incorporation of foreign genetic elements, including phages, plasmids, and other mobile elements that could disrupt cellular integrity [87]. The presence of this system has been associated with a reduced incidence of prophages, supporting its role in limiting phage infection and consequently restricting their genomic integration. In the genome of LP309, two distinct CRISPR-Cas systems were detected: type I-E and type II-A. The type I-E locus comprised 7 *Cas* genes (*cas1*, *cas2*, *cas3*, *cse2*, *cas7*, *cas5*, and *cas6*), whereas the type II-A operon included 4 *Cas* genes (*cas9*, *cas1*, *cas2*, and *csn2*). This genomic organization closely mirrors that described for other *L. pentosus* strains, including MP-10 and LPG1 [18, 19]. Additionally, up to 4 CRISPR arrays were identified using the CRISPRcasFinder tool and validated through CRISPRdb. These arrays consist of alternating direct repeats and spacer sequences, the latter derived from fragments of exogenous DNA acquired from previous encounters with plasmids or bacteriophages [87]. Such a system likely contributes to the genomic stability of LP309 by limiting the horizontal acquisition of elements associated with antibiotic resistance, virulence, or other potentially deleterious traits.

Mobile genetic elements (MGEs) encompass a variety of components, including bacteriophages, insertion sequences IS, transposons, integrons, and genomic islands (GIs). During the lysogenic cycle, bacteriophages can integrate into the bacterial genome, becoming prophages [88]. Such integrations are commonly observed in the genomes of *Lactobacillaceae*, particularly in probiotic strains [89, 90]. The number of prophages detected in LP309 is comparable to that reported for other *L. pentosus* as L33 and LPG1 strains [19, 58]. Three prophages were identified in LP309, integrated into chromosomal segments 2 and 3, which are recurrent insertion sites observed in *Lactobacillaceae* strains [91]. The same study also demonstrated that prophages can be used as taxonomic markers for *Lactobacillaceae* species. Beyond their role in lysogeny, prophages can contribute novel genetic material to the host genome, potentially conferring new traits [92], including antimicrobial resistance or virulence determinants [93]. However, in LP309, no genes associated with drug resistance or virulence were detected within the predicted prophages. Taxonomic classification of each latent phage was attempted, but due to the recent reclassification of bacteriophage families [94], all three prophages remained as unclassified *Caudoviricetes*.

A total of 133 insertion sequences were predicted in LP309. These elements can disrupt gene function by inserting into coding regions and interfering with transcription [95], but they also contribute to genomic plasticity and evolution [96]. When compared to other *Lactobacillaceae*, such as *Lactobacillus helveticus* CNRZ32 with 179 complete IS elements [97], LP309 harbors a lower IS count. This reduced number may be advantageous, as it likely results in fewer gene disruptions across the genome.

Genomic islands (GIs) are well known for carrying genes that provide selective advantages to their bacterial hosts [98]. Beneficial GIs can enhance genome diversification and drive bacterial evolution, as demonstrated in multiple species [99]. In LP309, four GIs were identified, mostly located near the chromosomal termini. Among the genes within these islands, the second GI contained a cluster encoding a siderophore transport system, crucial for iron acquisition under iron-limited conditions. This cluster included *yusV*, which encodes an ATP-binding protein of an ABC transporter for siderophores; *feuB*, which encodes the permease subunit of the FeuABC system; and associated genes forming the transmembrane components of the iron-siderophore uptake machinery. Such adaptive traits are particularly advantageous in environments where iron is scarce, and previous studies have classified siderophore-associated GIs as adaptive islands [100].

Among MGEs, plasmids are particularly relevant because of their ability to introduce new functionalities into bacterial genomes, including the production of EPSs or γ-aminobutyric acid (GABA) [101, 102]. In LP309, 8 plasmids were predicted by RFplasmid, each corresponding to a distinct contig. Subsequent sequence comparisons against plasmid databases from *L. plantarum* and *L. pentosus* confirmed their plasmid identity, as each contig aligned with a different reference plasmid. All plasmids exhibited over 50% coverage and greater than 94% sequence identity, with two plasmids showing complete coverage and above 99% identity. These results suggest a high conservation of functional elements and indicate the potential for horizontal gene transfer. Notably, LP309 is among the *L. pentosus* strains with the highest plasmid content reported, alongside LB-1, CNRZ1547, and KW2. Closing the genome using a hybrid assembly approach allowed us to estimate the relative copy number of each plasmid present in the sequenced population of *L. pentosus* LP309. The observed variation in plasmid abundance, ranging from single-copy plasmids to the high-copy-number plasmid LP309pl7, is consistent with the presence of distinct replication control mechanisms operating across different replicon types. Low-copy plasmids are typically associated with stringent replication control and often encode partitioning systems to ensure stable inheritance, whereas high-copy plasmids generally rely on relaxed replication control and increased copy number to maintain stability [103]. Automatic annotation combined with manual curation of plasmid LP309pl7 predicted that the majority of its genes (5 out of 8) are involved in replication and mobilization processes, while the remaining genes encode proteins of unknown function. Accordingly, this plasmid appears to represent a cryptic plasmid whose primary role is likely maintenance within the population rather than conferring an evident phenotype based on in silico analyses. Furthermore, analysis of genes involved in plasmid replication and transfer revealed a heterogeneous plasmid mobilization landscape within LP309. While only LP309pl1 was predicted to be potentially conjugative based on the presence of a conjugal transfer protein (Table S4), several plasmids were classified as mobilizable due to the presence of relaxases and mobilization-related proteins. This configuration suggests that horizontal gene transfer may occur predominantly through mobilization events mediated by conjugative elements rather than through autonomous conjugation [104]. In contrast, the absence of mobilization-associated genes in plasmids LP309pl6 and LP309pl8 suggests a reduced potential for horizontal transfer, supporting their role as stable genetic elements maintained within the host genome. Overall, the coexistence of plasmids with distinct copy numbers and transfer capabilities highlights the complex plasmid architecture of LP309 and underscores the potential contribution of plasmids to its technological robustness and ecological adaptation. Functionally, these plasmids encode traits that may enhance the strain’s ecological fitness. Specifically, plasmid 3 harbors a bacteriocin-associated region predicted as a bovicin 255–like locus. Although bovicin 255 has been reported to exhibit antimicrobial activity against *L. monocytogenes*, *C. perfringens* and *Salmonella enterica* subsp. *enterica* ser. *Typhi* in other *L. pentosus* strains [70], manual inspection of the LP309 locus revealed that it lacks the core biosynthetic genes required for lantibiotic production. Therefore, no functional bacteriocin activity is inferred for LP309 based on genomic data alone. Additionally, plasmid 1 carries a predicted EPS biosynthetic cluster. EPS production facilitates biofilm formation, as the polysaccharide matrix contributes to surface adherence and protection in planktonic states, enhancing environmental resilience [82, 84]. In fact, this plasmid, as shown in Table 5, shares a high similarity with a plasmid from *L. plantarum* L75. Recently, Su et al. [105] studied the EPS produced by this strain to evaluate its effects on oat silage fermentation, antioxidant capacities, and microbial community dynamics. Therefore, it is plausible that both *L. plantarum* L75 and *L. pentosus* LP309 produce EPS with similar functions.

## Conclusions

The integration of hybrid sequencing technologies with comprehensive bioinformatic analyses enabled a high-resolution characterization of the *L. pentosus* LP309 genome. The hybrid assembly strategy effectively overcame the limitations of single-platform technologies, producing a robust genome assembly. This approach revealed key genomic features related to functionality, safety, and adaptability, supporting its potential use as a starter culture for vegetables and/or plant-derived probiotic, based on in silico predictions that require experimental validation.

## Supplementary Information

Supplementary Material 1.

## Data Availability

ENA repository access number PRJEB94968. BioSample number: Illumina reads (SAMEA118838869), PacBio reads (SAMEA120476442), MinION fast reads (SAMEA120476443), and MinION sup reads (SAMEA120476444).
